# Self-reported domain-specific and accelerometer-based physical activity and sedentary behaviour in relation to psychological distress among an urban Asian population

**DOI:** 10.1186/s12966-018-0669-1

**Published:** 2018-04-05

**Authors:** A. H. Y. Chu, R. M. van Dam, S. J. H. Biddle, C. S. Tan, D. Koh, F. Müller-Riemenschneider

**Affiliations:** 1 Saw Swee Hock School of Public Health, National University of Singapore, Singapore, 117549 Singapore; 2 Department of Nutrition, Harvard School of Public Health, Boston, MA 02115 USA; 3 Physically Active Lifestyles (PALs) Research Group, Institute for Resilient Regions, University of Southern Queensland, Springfield Central, Ipswich, Australia; 4 PAPRSB Institute of Health Sciences, Universiti Brunei Darussalam, Jalan Tungku Link, Gadong, BE1410 Brunei Darussalam; 5 Institute for Social Medicine, Epidemiology and Health Economics, Charité University Medical Centre, 10117 Berlin, Germany

**Keywords:** Physical activity, Self-report, Accelerometry, Mental health, Adult

## Abstract

**Background:**

The interpretation of previous studies on the association of physical activity and sedentary behaviour with psychological health is limited by the use of mostly self-reported physical activity and sedentary behaviour, and a focus on Western populations. We aimed to explore the association of self-reported and devise-based measures of physical activity and sedentary behaviour domains on psychological distress in an urban multi-ethnic Asian population.

**Methods:**

From a population-based cross-sectional study of adults aged 18–79 years, data were used from an overall sample (*n* = 2653) with complete self-reported total physical activity/sedentary behaviour and domain-specific physical activity data, and a subsample (*n* = 703) with self-reported domain-specific sedentary behaviour and accelerometry data. Physical activity and sedentary behaviour data were collected using the Global Physical Activity Questionnaire (GPAQ), a domain-specific sedentary behaviour questionnaire and accelerometers. The Kessler Screening Scale (K6) and General Health Questionnaire (GHQ-12) were used to assess psychological distress. Logistic regression models were used to calculate odds ratios (ORs) and 95% confidence intervals, adjusted for socio-demographic and lifestyle characteristics.

**Results:**

The sample comprised 45.0% men (median age = 45.0 years). The prevalence of psychological distress based on the K6 and GHQ-12 was 8.4% and 21.7%, respectively. In the adjusted model, higher levels of self-reported moderate-to-vigorous physical activity (MVPA) were associated with significantly higher odds for K6 (OR = 1.47 [1.03–2.10]; p-trend = 0.03) but not GHQ-12 (OR = 0.97 [0.77–1.23]; p-trend = 0.79), when comparing the highest with the lowest tertile. Accelerometry-assessed MVPA was not significantly associated with K6 (p-trend = 0.50) nor GHQ-12 (p-trend = 0.74). The highest tertile of leisure-time physical activity, but not work- or transport-domain activity, was associated with less psychological distress using K6 (OR = 0.65 [0.43–0.97]; p-trend = 0.02) and GHQ-12 (OR = 0.72 [0.55–0.93]; p-trend = 0.01). Self-reported sedentary behaviour was not associated with K6 (p-trend = 0.90) and GHQ-12 (p-trend = 0.33). The highest tertile of accelerometry-assessed sedentary behaviour was associated with significantly higher odds for K6 (OR = 1.93 [1.00–3.75]; p-trend = 0.04), but not GHQ-12 (OR = 1.34 [0.86–2.08]; p-trend = 0.18).

**Conclusions:**

Higher levels of leisure-time physical activity and lower levels of accelerometer-based sedentary behaviour were associated with lower psychological distress. This study underscores the importance of assessing accelerometer-based and domain-specific activity in relation to mental health, instead of solely focusing on total volume of activity.

**Electronic supplementary material:**

The online version of this article (10.1186/s12966-018-0669-1) contains supplementary material, which is available to authorized users.

## Background

Psychological distress, also referred to as stress or emotional distress, is a type of non-specific mental health problem commonly used to describe a range of negative parameters such as self-deprecation, irritability, anxiety, depression and social disengagement [1]. It is the body’s response to external stressors [2]. Individuals with psychological distress may show symptoms such as lacking enthusiasm, having sleep problems, feeling downhearted or “blue”, feeling hopeless in life, getting “emotional”, and feeling like crying [3]. A nationwide study of 6616 Singaporean adults reported that 12.0% of the population had at least one lifetime anxiety, affective or alcohol use disorder [4]. The prevalence of common mental health problems (i.e. associated with mood, anxiety and substance use disorders) worldwide among adults is 17.6% within the past 12 months and 29.2% across the lifespan; and women have a higher prevalence for mood or anxiety disorder than men [5]. Traditionally, the assessment of psychological distress has been performed using self-report measures [6]. Two of the most widely used self-reported screening tools for psychological distress are the Kessler 6 (K6) [7] and GHQ-12 (General Health Questionnaire-12) [8], which have been translated into different languages and extensively validated in general and clinical populations [9–13]. The K6 and GHQ-12 have slightly different properties in assessing psychological health outcomes with regards to the reference period, in which the K6 assessed respondents’ feelings for the past 30 days, while the GHQ-12 assessed if respondents’ “present state” differed from their “usual state”.

Regular physical activity [14–18] and less sedentary behaviour [19–21] have been shown to associate with better mental health. Several biological mechanisms of the effect of physical activity on psychological states have been proposed, including a reduction in stress hormones (e.g. cortisol, adrenaline), stimulation of mood-elevating chemicals (e.g. endorphins, serotonin), and an increase in the release of proteins with neuroprotective functions and antidepressant role (e.g. brain-derived neurotrophic factor) [22–25]. Psychological mechanisms that occur with physical activity have also received considerable attention. For example, the distraction theory suggests that diversion from painful stimulus or unpleasant events following exercise leads to improved affect [26]. The self-efficacy theory suggests that confidence in one’s ability to perform physical activity is strongly associated with the actual performance [27]. Other hypotheses including the mastery theory and social interaction hypothesis were also suggested to be responsible for the improved effects on mental health [28, 29].

To date, many studies investigating these associations have involved self-reported physical activity or sedentary behaviour measures, which are subject to recall and response bias when compared to so-called ‘objective’ measures using wearable devices [30–32]. A relatively small body of literature has evaluated the associations of accelerometer-based physical activity/sedentary behaviour and psychological distress, and a majority of previous studies were from Western countries. Also, accelerometer-based data has produced different findings to self-report data. For example, Hamer et al. [33] found that lower levels of distress were associated with higher levels of self-reported physical activity but not accelerometer-based physical activity. For sedentary behaviour, on the other hand, there was evidence showing that both accelerometer-based and self-reported sedentary behaviour were associated with higher prevalence of psychological distress in the UK population [33]. Since the evidence for the associations of accelerometer-based physical activity and sedentary behaviour with mental health seems inconsistent, further investigation is warranted.

Moreover, given the complex nature of physical activity and sedentary behaviour, quantifying physical activity or sedentary behaviour into a single value may be overly simplistic. Previous research in this area has focused primarily on leisure-time physical activity. Few studies have investigated the association of domain-specific physical activity/sedentary behaviour and psychological distress among adults [34]. While leisure-time physical activity was found to provide health benefits, the associations of work-domain physical activity and health have been conflicting [35]. Individuals’ motivation, preferences or obligations (e.g. voluntary physical activity during leisure-time vs. participation of work-domain physical activity due to physically demanding work) for physical activity performed across different domains could elicit variability in psychological stress responses. As for sedentary behaviour, little research has been done on assessing the association of sedentary behaviour in different contexts (i.e. occupational, motorized transportation, and leisure-time). For example, a study has examined non-occupational sedentary behaviour among 2707 Australian working adults [20], showing that TV viewing, computer use, and total non-occupational sedentary behaviour were associated with worse mental health in women whereas in men, only computer use was associated with poor mental well-being.

Taken together, the interpretation of previous studies is limited by: i) the majority of research measured total volume of physical activity (or sedentary behaviour), ii) mixed findings in a limited number of studies comparing association between physical activity (or sedentary behaviour) and psychological distress using accelerometer-based and self-reported physical activity (or sedentary behaviour), iii) a focus mainly on Western populations. Using a nationally representative sample from Singapore, an urban Asian population, we aimed to assess whether the association between physical activity (or sedentary behaviour) and psychological distress is comparable when using accelerometer-based and self-reported physical activity (or sedentary behaviour). We seek to explore the associations of physical activity (or sedentary behaviour) and mental health using two different psychological distress scales. Second, we aimed to expand on the literature by comprehensively examining the extent to which total and domain-specific physical activities (or sedentary behaviour) are associated with psychological distress.

## Methods

### Study design and participants

The Singapore Health 2 (SH2) is a nationally representative cross-sectional survey of the physical, mental and self-rated overall health of the Singapore residents. The study was conducted between April 2014 and April 2015.

Participants were randomly selected through multistage stratified cluster sampling. Stratification was based on geographical regions. The first-stage sampling included 32,100 randomly selected household addresses with at least 1 resident aged 18–79 years from the National Database on Dwellings maintained by the Department of Statistics in Singapore. Second-stage sampling included 15,000 randomly selected household addresses from the sampling frame.

Inclusion criteria:

1. Singaporeans or permanent residents,
2. aged 18 to 79 years at entry,
3. stayed at least 4 days per week in the household and were staying in the household for 3 months or longer after the time of enumeration.

Exclusion criteria:

1. household members who had severe mental retardation and mental illness,
2. stroke or injury resulting in loss of speech, were bedridden, or wheelchair-bound,
3. pregnant women.

Out of the 7749 eligible households, a total of 2686 adults participated in the SH2 (yielding a response rate of 35%) and provided self-reported total/domain-specific physical activity, and total sedentary behaviour data. A total of 895 individuals agreed to participate in a dedicated physical activity sub-study for additional assessments of accelerometer-based physical activity and sedentary behaviour, and self-reported domain-specific sedentary behaviour.

The study was approved by the National University of Singapore Institutional Review Board (NUS IRB: reference 13–512). Written informed consent was obtained from all participants. After providing informed consent, participants were interviewed by trained interviewers. All study interviewers were briefed extensively on the study methodologies and underwent rigorous training in the study procedures assigned to them. This was to ensure strict compliance with the standards and procedures of the study. Training of interviewers on consent taking, interviewing and advising on the preparation for the physical examination appointment was conducted by experienced staff from the Saw Swee Hock School of Public Health.

### Demographics and clinical disease characteristics

Participants who consented to participate in this study completed a questionnaire through a face-to-face interview. Socio-demographics characteristics (i.e. age, gender, ethnicity, educational level, marital status, monthly household income and employment status), smoking status, alcohol drinking and body mass index (BMI) were collected from the interview. Study participants were also asked whether they ever had the following chronic diseases: asthma, cancer, diabetes mellitus, heart attack, and stroke.

### Physical activity and sedentary behaviour measures

#### Self-reported physical activity

Self-reported duration and frequency of physical activity over a typical week were assessed using the Global Physical Activity Questionnaire (GPAQ) previously validated in our population [36]. The GPAQ was developed by the World Health Organization (WHO) in 2002 as part of the WHO STEPwise Approach to Chronic Disease Risk Factor Surveillance (STEPS) for physical activity and sedentary behaviour surveillance [37]. The GPAQ comprises 15 questions to capture the intensity, frequency, and duration of physical activity in three domains (work-, transportation-, and leisure-time), as well as an additional question on sedentary behaviour. Weekly moderate-to-vigorous intensity physical activity (MVPA) and domain-specific physical activity across three domains including work (which also comprises household activity), transportation and leisure-time were collected.

#### Self-reported sedentary behaviour

Total sedentary time was calculated based on: (i) a single-item sitting question, “How much time do you usually spend sitting or reclining on a typical day?” adopted from the GPAQ, and (ii) a weighted sum of daily domain-specific sedentary time (weekday sitting minutes*5/7 + weekend day sitting minutes*2/7) adapted from existing and established domain-specific sedentary behaviour questionnaires [38–41]. The single-item sitting question was asked to all SH2 participants. Participants in the physical activity sub-study were asked the following additional items on domain-specific sedentary behaviour during a typical week:

1. sitting time spent working (in the office, school or home),
2. sitting during transportation (motor vehicle),
3. sitting while watching television (TV), DVD/video viewing or screen viewing, such as watching videos (i.e. YouTube, online video), internet surfing, using social media, playing electronic games on any media device e.g. on computer, tablet or mobile phone during leisure-time only.

Time spent sitting in each domain was summarized as hours per day (h/d), and capped at 24-h per day (adapted from Rosenberg et al.’s [40] data cleaning protocol). Respondents with invalid summaries of time spent were removed from all analyses.

### Accelerometer-based physical activity and sedentary behaviour

Participants from the sub-study were invited to wear the triaxial ActiGraph wGT3X-BT accelerometer (ActiGraph, LLC, Pensacola, Florida, USA) placed on the right hipbone for seven consecutive days, at least 10 h/d [42, 43]. The accelerometers were worn after completing the questionnaires. The accelerometers were initialized at a sampling rate of 30 Hz for data collection and distributed to the participants in person by trained interviewers. Participants were allowed to wear the accelerometers overnight or remove it during bedtime and re-attach it upon waking up in the morning. They were allowed to remove their accelerometers during water-based activities (e.g. showering, swimming, etc.). An instruction sheet containing details of how to wear the accelerometers and removing them during water-based activities was provided to the participants. Log sheets were given to participants to record the dates and times when their accelerometers were worn and removed. Accelerometry data were downloaded and integrated into 60-s epochs, sleep scored, wear time validated, processed and scored for physical activity and sedentary behaviour variables using ActiLife software (Version 6).

### Visual inspection of bedtime and wake time

Although participants were allowed to remove the accelerometers prior to bedtime, some participants wore them 24 h a day. In the data scoring analysis, bedtime may be inadvertently identified as sedentary behaviour time due to very low-intensity activities. Therefore, visual inspection of ‘in-bed’ and ‘out-of-bed’ period was graphically flagged on a minute-by-minute, day-to-day, participant-by-participant basis by two trained researchers.

The protocol to screen for bedtime wearing was by identifying the activity counts which:

1. began with a period of low activity counts (approximately ≤50% of the participant’s average daytime activity level) to zeros, lasting at least 5–10 consecutive minutes,
2. persisted to have activity counts that did not rise approximately > 50% of average daytime activity level for more than 5 consecutive minutes within a 3-h period.

Short occurring periods of activity count rise within the visually identified bedtime were allowed if the total duration was < 10 min. Wake time was identified as activity counts of > 0 lasting for at least 10–15 consecutive minutes. The sleep scoring method used in this study was partly based on Kinder et al.’s [44] approach to visually analyse waist-worn accelerometer data. In addition, the self-reported bedtime and wake times from participants were considered in the visual inspection for bedtime when there is uncertainty. From the visual inspection, all sleep periods were graphically flagged as non-wear time, and subsequently, wear time validation was performed.

### Wear time validation and scoring

Non-wear time was defined as 90-consecutive minutes of zero accelerometer counts per min (CPM), and the artefactual movements detection was set to allow interruptions up to a 2-min interval accompanied by either up or downstream 90-min consecutive zero CPM window [45]. The criterion for determining valid monitoring days was having ≥4 days with ≥10 h/d of waking hours in a 24-h period [46].

Using validated vector magnitude cut-points based on the built-in algorithm of the ActiLife software, the following summary metrics were calculated: sedentary behaviour (< 150 CPM) and MVPA (≥2690 CPM) [47, 48]. Accelerometry-based MVPA was analysed by accumulated time in bouts of ≥10-min.

### Psychological distress

Psychological distress was measured using the Kessler Screening Scale (K6) [7], with the General Health Questionnaire (GHQ-12) [8] scale as an additional psychological dimension.

Using the K6, the frequency of non-specific psychological distress experienced by study participants during the last 30 days was measured. The K6 consists of six questions asking how often one felt: (i) nervous, (ii) hopeless, (iii) restless or fidgety, (iv) so depressed that nothing could cheer one up, (v) that everything was an effort, (vi) and worthless. A value of 1, 2, 3, 4 or 5 was assigned to each five Likert-type response category for each question: “none of the time”, “a little of the time”, “some of the time”, “most of the time”, or “all of the time”, respectively. Responses were summed with a score range of 6–30. Based on previous studies, the summed scores can be classified into three categories: i) no or low psychological distress: 6–13; ii) moderate psychological distress: 14–18; and iii) high psychological distress: 19–30 [49]. Participants were subsequently classified into two groups: no-to-low distress (≤13) vs. moderate-to-high distress only (≥14). Participants with incomplete responses to the questionnaires were excluded from the analysis.

The GHQ-12 measures psychological distress by asking if the respondents’ current state differs from their usual state. Thus, the GHQ-12 is sensitive to short-term psychological conditions. The GHQ-12 consists of 12 questions in the contexts of: concentration, losing much sleep, feeling useful, capable of making decisions, under stress, inability to overcome difficulties, enjoy normal activities, able to face up to problems, feeling unhappy and depressed, losing confidence, feelings of worthlessness, and feeling reasonably happy. The original Goldberg’s scoring method was used, with each response category of “not at all”, “no more than usual”, “rather more than usual”, “much more than usual” corresponding to a value of 0–0–1-1, respectively. A respondent’s total score (summed across all questions) ranges from 0 to 12. Using a cut-off of ≥2 to detect psychological distress was validated in Singapore [50] and by the WHO [8]. The GHQ-12 was used as a one-dimensional scale in the current study to keep its originality and simplicity in our analysis [12].

### Data analysis

Descriptive analyses were presented as median (interquartile range; IQR) for continuous variables as they were not normally distributed, and counts and percentages for categorical variables. Differences in the socio-demographic and clinical disease characteristics between psychological distress levels in the overall sample and physical activity sub-study were tested using Mann-Whitney U test for continuous variables and chi-square test for categorical variables. Logistic regression was used to model psychological distress, such that self-reported and accelerometer-based physical activity/sedentary behaviour variables were exposures categorized into tertiles. For the categorization of self-reported domain-specific physical activity, a high proportion of participants reported zero activity per week in work- and leisure-time domains (57.9% and 50.8%, respectively). Therefore, for the categorization of tertiles across all domain-specific physical activity (work-, transport-, and leisure-time domains), all zero values were defined as a separate category (1st tertile). The remaining values were then stratified into two categories based on a median split to form a total of three categories (2nd and 3rd tertile).

In Model 1, the univariable association was examined. In Model 2, adjustments were made for age, gender, ethnicity, educational level, employment status, BMI, marital status, smoking status, alcohol drinking, the presence of at least one chronic disease; with total sedentary behaviour added in the physical activity analyses, and vice versa. We reported the association between the exposures and outcomes with the odds ratio (OR) and the corresponding 95% confidence interval (CI). When we explored the relationships of moderate- and vigorous-intensity physical activity with psychological distress, the associations were not significant (data not presented). To test for linear trends across categories, we modelled physical activity and sedentary behaviour as continuous variables in their original form. We also tested whether gender, ethnicity, and age could be effect modifiers in the association between the exposures and outcomes. Assumptions for the logistic regression analyses were met and multicollinearity among independent variables was investigated (variance inflation factor [VIF] values < 1.97) [51]. The significance level was set at 0.05. The statistical analysis was performed using the Stata Statistical Software version 14 (College Station, TX: StataCorp LP).

## Results

### Study participants

Participant characteristics are presented in Table 1. A total of 2653 participants who provided valid self-reported total physical activity and sedentary behaviour, domain-specific physical activity data and psychological distress measures were included in the analysis. The majority of participants were women (55.0%), of Chinese ethnicity (66.7%), currently married (64.1%), had at least pre-tertiary education (69.8%), non-smokers (70.6%), non-alcohol drinkers (54.0%), with normal BMI (44.3%), and employed (74.5%). Approximately a fifth of participants (20.4%) reported ever having at least one chronic disease. A total of 703 participants who provided complete information on domain-specific sedentary behaviour, valid accelerometer-based physical activity/sedentary behaviour data, and psychological distress measures were included in the sub-study. Participants from the physical activity sub-study as compared with those in the main SH2 study were statistically significantly different in gender, educational level, stroke, and presence of at least one chronic disease (i.e. asthma, cancer, diabetes mellitus, heart attack, and stroke).

**Table 1:** Participants’ characteristics in SH2 study and physical activity sub-study

	Overall (*n* = 2653)		Subsample (*n* = 703)		*p*-value*
	n	(%)	n	(%)	
Age (Med, IQR)	45.0 (34.0–58.0)		46.0 (35.0–58.0)		0.50
Age Group					0.47
18–29	433	16.3	115	16.4	
30–39	534	20.1	135	19.2	
40–49	592	22.3	162	23.0	
50–59	515	19.4	154	21.9	
60–79	579	21.8	137	19.5	
Gender					0.04
Men	1193	45.0	285	40.5	
Women	1460	55.0	418	59.5	
Ethnicity					0.74
Chinese	1770	66.7	469	66.7	
Indian	402	15.2	97	13.8	
Malay	387	14.6	110	15.6	
Others	94	3.5	27	3.8	
Marital status					0.11
Not married^a^	952	35.9	275	39.1	
Married	1701	64.1	428	60.9	
Education level					< 0.01
Secondary & below	800	30.2	161	22.9	
Pre-tertiary	1210	45.6	356	50.6	
University & above	643	24.2	186	26.5	
Employment status					0.18
Unemployed	677	25.5	162	23.0	
Employed	1976	74.5	541	77.0	
BMI, kg/m^2^ (Med, IQR)	23.6 (21.0–26.5)		23.7 (21.1–26.6)		0.33
BMI category					0.33
≤ 22.9 Normal	1174	44.3	291	41.4	
≥ 23.0 & ≤27.4 Overweight	978	36.9	279	39.7	
≥ 27.5 Obese	501	18.9	133	18.9	
Smoking status					0.22
Never smoker	1873	70.6	513	73.0	
Ever smoker	780	29.4	190	27.0	
Alcohol drinking					0.08
Non-drinker	1433	54.0	360	51.2	
Irregular drinker	984	37.1	291	41.4	
Regular drinker	236	8.9	52	7.4	
Psychological distress					
K6 score ≥ 14	224	8.4	69	9.8	0.25
K6 (Med, IQR)	7.0 (6.0–10.0)		8.0 (6.0–10.0)	0.05	
GHQ-12 score ≥ 2	577	21.7	175	24.9	0.08
GHQ-12 (Med, IQR)	0 (0–1.0)		0 (0–1.0)		0.57
Asthma	281	10.6	69	9.8	0.55
Cancer	52	2.0	16	2.3	0.60
Diabetes mellitus	235	8.9	52	7.4	0.22
Heart attack	34	1.3	9	1.3	0.99
Stroke	31	1.2	1	0.1	0.01
Presence of at least one disease	172	6.5	136	19.3	< 0.01

*p-value: Test of significance between overall sample and subsample

^a^Single, divorced or widowed

### Psychological distress

The prevalence of psychological distress based on the K6 was 8.4% and based on the GHQ-12 was 21.7% (Table 1). Characteristics of participants according to the K6 psychological distress score in each overall sample and subsample are presented in Additional file 1: Table S1, those who had moderate-to-high distress tended to be younger, not married, had asthma and had at least one chronic disease in the overall sample (*p*-value< 0.05). Differences in the characteristics between K6 psychological distress levels in the subsample presented similar findings for the aforementioned variables except for educational level, employment status, and smoking status.

Based on the GHQ-12, those who were distressed tended to be younger, married, regular alcohol drinkers, had asthma or at least one chronic disease in the overall sample (*p*-value< 0.05) (Additional file 1: Table S2). Differences in the characteristics between GHQ-12 psychological distress levels in the subsample presented similar findings for the aforementioned variables except for alcohol drinking and had at least one chronic disease.

### Total physical activity, sedentary behaviour and psychological distress

#### Self-reported physical activity and sedentary behaviour

Table 2 presents the association of total MVPA and sedentary behaviour with psychological distress. In comparison with the lowest tertile, the unadjusted and adjusted ORs suggest that higher levels of self-reported MVPA were associated with higher psychological distress using the K6 (adjusted OR = 1.47; 95% CI = 1.03–2.10), but not the GHQ-12 (adjusted OR = 0.97; 95% CI = 0.77–1.23). There was evidence of a linear trend between MVPA and higher psychological distress using the K6 (p for trend = 0.03). Self-reported sedentary behaviour was not significantly associated with psychological distress using the K6 or GHQ-12 in the adjusted model. No significant effect modification/potential interaction by gender, ethnicities and age group was observed.

**Table 2:** Physical activity and sedentary behaviour by self-report and accelerometry in relation to psychological distress

		K6						GHQ-12					
		Model 1^a^			Model 2^b^			Model 1^a^			Model 2^b^		
Self-reported (*n* = 2653)	n	OR	95% CI		OR	95% CI		OR	95% CI		OR	95% CI	
MVPA (min/wk)													
T1 (≤200.0)	903	Ref.			Ref.			Ref.			Ref.		
T2 (> 200.0 & ≤520.0)	874	1.20	0.84	1.72	1.11	0.77	1.60	0.85	0.68	1.06	0.82	0.65	1.04
T3 (> 520.0)	876	1.64	1.17	2.30	1.47	1.03	2.10	0.99	0.80	1.24	0.97	0.77	1.23
p for trend		0.004			0.03			0.95			0.79		
Sedentary behaviour (h/d)													
T1 (≤4.0)	955	Ref.			Ref.			Ref.			Ref.		
T2 (> 4.0 & ≤8.0)	1112	1.18	0.87	1.62	1.20	0.86	1.66	1.09	0.88	1.34	1.04	0.83	1.30
T3 (> 8.0)	586	1.02	0.70	1.50	0.94	0.62	1.42	1.28	1.00	1.64	1.15	0.88	1.50
p for trend		0.77			0.90			0.05			0.33		
Accelerometry (*n* = 703)	n	OR	95% CI		OR	95% CI		OR	95% CI		OR	95% CI	
MVPA (min/wk)													
T1 (≤32.2)	235	Ref.			Ref.			Ref.			Ref.		
T2 (> 32.2 & ≤115.5)	237	1.21	0.65	2.26	1.34	0.67	2.68	1.23	0.82	1.86	1.23	0.79	1.91
T3 (> 115.5)	231	1.30	0.70	2.42	1.28	0.62	2.64	0.95	0.62	1.46	0.92	0.58	1.47
p for trend		0.40			0.50			0.83			0.74		
Sedentary behaviour (h/d)													
T1 (≤7.3)	235	Ref.			Ref.			Ref.			Ref.		
T2 (> 7.3 & ≤8.9)	234	0.78	0.39	1.57	0.82	0.38	1.75	0.70	0.45	1.09	0.84	0.53	1.34
T3 (> 8.9)	234	2.00	1.11	3.61	1.93	1.00	3.75	1.36	0.91	2.05	1.34	0.86	2.08
p for trend		0.01			0.04			0.12			0.18		

*OR* odds ratio, *CI* confidence interval, *MVPA* moderate-to-vigorous physical activity

^a^Unadjusted odds

^b^Adjusted for age, gender, ethnicity, marital status, education, employment status, BMI, smoking status, alcohol drinking, presence of at least 1 disease and mutually adjusted for physical activity and sedentary behaviour, respectively

### Accelerometer-based physical activity and sedentary behaviour

There was no significant linear trend between accelerometer-based MVPA and psychological distress using the K6 or GHQ-12 (Table 2). However, the highest tertile of accelerometer-based sedentary behaviour had borderline significance with higher psychological distress using the K6 (adjusted OR = 1.93; 95% CI = 1.00–3.75, p for trend = 0.04), whereas no significant association was reported for GHQ-12 psychological distress.

### Domain-specific physical activity, sedentary behaviour and psychological distress

Within physical activity domains, there was a significant linear trend between work-domain physical activity and K6 psychological distress (Table 3). The highest tertile of work-domain physical activity was significantly associated with higher odds of psychological distress using the K6 (adjusted OR = 1.75; 95% CI = 1.23–2.50; p for trend = 0.003), but not the GHQ-12 (adjusted OR = 1.14; 95% CI = 0.88–1.47; p for trend = 0.29). No significant linear trend was observed between transport-domain physical activity and psychological distress by any scales. In contrast, the highest tertile of leisure-time physical activity was associated with lower odds for K6 psychological distress (adjusted OR = 0.65; 95% CI = 0.43–0.97; p for trend = 0.02). Similarly, increasing activity across tertiles of leisure-time physical activity was associated with decreasing odds for GHQ-12 psychological distress (Middle tertile: adjusted OR = 0.77; 95% CI = 0.61–0.96, highest tertile: adjusted OR = 0.72; 95% CI = 0.55–0.93; p for trend = 0.01). Within sedentary behaviour domains, none of the adjusted domain-specific sedentary behaviour variables was significantly associated with psychological distress.

**Table 3:** Domain-specific physical activity and sedentary behaviour by self-report in relation to psychological distress

		K6						GHQ-12					
		Model 1^a^			Model 2^b^			Model 1^a^			Model 2^b^		
Physical activity (*n* = 2653)	n	OR	95% CI		OR	95% CI		OR	95% CI		OR	95% CI	
Work (min/wk)^c^													
T1 (=0)	1536	Ref.			Ref.			Ref.			Ref.		
T2 (> 0 & ≤360.0)	595	1.24	0.88	1.75	1.19	0.83	1.70	1.09	0.87	1.37	1.08	0.85	1.37
T3 (> 360.0)	522	1.74	1.26	2.43	1.75	1.23	2.50	1.10	0.87	1.40	1.14	0.88	1.47
p for trend		0.001			0.003			0.36			0.29		
Transport (min/wk)^c^													
T1 (=0)	591	Ref.			Ref.			Ref.			Ref.		
T2 (> 0 & ≤210.0)	1305	1.39	0.96	2.03	1.26	0.85	1.85	0.83	0.66	1.05	0.78	0.61	1.00
T3 (> 210.0)	757	1.40	0.93	2.10	1.22	0.80	1.86	0.87	0.67	1.12	0.83	0.64	1.08
p for trend		0.14			0.43			0.32			0.21		
Leisure-time (min/wk)^c^													
T1 (=0)	1347	Ref.			Ref.			Ref.			Ref.		
T2 (> 0 & ≤120.0)	769	0.81	0.58	1.11	0.70	0.50	1.00	0.86	0.69	1.07	0.77	0.61	0.96
T3 (> 120.0)	537	0.76	0.52	1.10	0.65	0.43	0.97	0.80	0.63	1.03	0.72	0.55	0.93
p for trend		0.09			0.02			0.06			0.01		
Sedentary behaviour (*n* = 703)	n	OR	95% CI		OR	95% CI		OR	95% CI		OR	95% CI	
Work (h/d)													
T1 (≤3.0)	271	Ref.			Ref.			Ref.			Ref.		
T2 (> 3.5 & ≤6.0)	209	0.83	0.45	1.53	0.87	0.44	1.74	1.03	0.68	1.57	1.01	0.69	1.73
T3 (> 6.0)	223	0.87	0.48	1.57	0.93	0.45	1.94	1.00	0.66	1.50	0.99	0.60	1.62
p for trend		0.62			0.84			1.00			0.97		
Transport (h/d)													
T1 (≤0.5)	255	Ref.			Ref.			Ref.			Ref.		
T2 (> 0.5 & ≤1.1)	228	1.18	0.64	2.2	0.84	0.42	1.65	1.19	0.79	1.78	1.22	0.79	1.88
T3 (> 1.1)	220	1.30	0.71	2.38	0.99	0.50	1.99	0.79	0.51	1.21	0.78	0.49	1.25
p for trend		0.40			0.99			0.32			0.33		
Leisure-time (h/d)													
T1 (≤1.6)	249	Ref.			Ref.			Ref.			Ref.		
T2 (> 1.6 & ≤3.0)	220	0.96	0.49	1.88	0.76	0.37	1.58	1.11	0.72	1.71	0.99	0.63	1.57
T3 (> 3.0)	234	1.81	1.01	3.27	1.28	0.65	2.52	1.56	1.02	2.38	1.41	0.90	2.21
p for trend		0.04			0.39			0.04			0.12		

*OR* odds ratio, *CI* confidence interval

^a^Unadjusted odds

^b^Adjusted for age, gender, ethnicity, marital status, education, employment status, BMI, smoking status, and alcohol drinking, presence of at least 1 disease and mutually adjusted for physical activity and sedentary behaviour, respectively

^c^T1 includes only zero values, T2 and T3 are grouped by median splitting the remaining values

The associations of domain-specific physical activity and psychological distress in the subsample are presented in Additional file 1: Table S3. The association between the highest tertile of work-domain physical activity and psychological distress using the K6 was of borderline significance in the subsample (adjusted OR = 1.97; 95% CI = 1.00–3.93; p for trend = 0.07). The middle tertile of leisure-time physical activity in the subsample was associated with lower odds for psychological distress using the K6 (adjusted OR = 0.47; 95% CI = 0.23–0.95; p for trend = 0.21). Similarly, the middle tertile of leisure-time physical activity was associated with lower odds for GHQ-12 psychological distress, with a statistically significant linear trend of borderline significance (adjusted OR = 0.64; 95% CI = 0.41–0.98; p for trend = 0.05). No significant linear trend was observed between transport-domain physical activity and psychological distress by any scales.

## Discussion

In this multi-ethnic adult Asian population, higher levels of self-reported MVPA were associated with a higher likelihood of psychological distress assessed by the K6 but not the GHQ-12. In contrast, total MVPA assessed by the accelerometers was not associated with psychological distress. Consideration of domain-specific physical activities revealed that this direct association between self-reported MVPA and psychological distress was mainly due to work-related physical activity, whereas higher leisure-time physical activity was associated with less psychological distress based on both the K6 and the GHQ-12. Self-reported total and domain-specific sedentary behaviour were not associated with psychological distress. However, higher levels of accelerometer-based total sedentary behaviour were associated with a greater likelihood of psychological distress, especially when assessed by the K6.

The difference in the association for self-reported total MVPA according to the K6 and the GHQ-12 in this study may in part be related to the different psychological measurement scales. As mentioned previously, the K6 reflects respondents’ psychological distress status of the past 30 days, whereas the GHQ-12 reflects their present psychological state relative to their usual state. Therefore, people with long-term chronic psychological health conditions may not respond optimally to the GHQ-12. This study finding was inconsistent with most previous studies [15, 30–33]. For example, the association between higher doses of MVPA and lower likelihood of psychological distress using the K6 has been described in a large population of Australian adults [32]. In a large representative sample of 78,886 U.S. adults aged ≥18-years from the 2009 Behavioral Risk Factor Surveillance System, individuals without serious psychological distress were more likely to self-report higher physical activity levels than those with serious psychological distress using the K6 [52]. Previous studies using the GHQ-12 also reported an association between higher levels of self-reported physical activity and less psychological distress in the Health Survey for England [33], the Scottish Health Survey [15, 30], and the Singapore’s 2010 National Health Survey [31]. The discrepancies between previous studies and ours may in part be due to the use of: i) different cut-off points for detecting psychological distress despite using the same scale (i.e. GHQ-12) [15, 30, 33], ii) different psychological distress scales (i.e. K10) [32], iii) or merely the diverse characteristics of study populations. Of note, despite the similarities between the study setting in Sloan et al.’s [31] and ours, the discrepancies in findings could partly be due to different classification of participants into categories (i.e. meeting or not meeting physical activity guidelines in Sloan et al.’s study vs. categorization into tertiles in our study).

Accelerometer-based MVPA was not associated with psychological distress in the current study. Such differences were also observed in Parker et al.’s study [53], of which accelerometer-based MVPA predicted psychological negative affectivity better than self-reports among older adults. Our study revealed substantial differences between accelerometer-based and self-report data (e.g. range of medium tertiles of time spent on MVPA based on self-report and accelerometry were > 200.0 & ≤520.0 min/week and > 32.2 & ≤115.5 min/week, respectively). Accelerometers are considered relatively accurate and reliable instrument to measure physical activity [54, 55], and thus may provide greater accuracy in reflecting the associations with psychological distress. Our finding is in line with Hamer et al.’s [33] study which did not observe any relationship between accelerometer-based MVPA and psychological distress. In contrast, previous studies found that higher levels of accelerometer-based MVPA were associated with less depressive symptoms using the Patient Health Questionnaire-9 in a sample of 2862 general adult population [56] and 708 older adults from the National Health and Nutrition Examination Survey (NHANES) 2005–2006 [57]. The use of different accelerometers (ActiGraph accelerometer model 7164) and cut-offs to categorize MVPA in Vallance et al. [56] (counts/min ≥1952) and Loprinzi’s studies [57] (counts/min ≥2020) could have also led to inconsistent results between accelerometer-based MVPA and psychological distress as seen in our study. Two previous studies in older Japanese adults also showed that higher levels of accelerometer-based physical activity assessed over a period of 1 year were associated with lower depressive scores [58] and stressful life events [59]. However, it should be noted that with different psychological measures, these results cannot be directly compared. Overall, the observed variations in health outcomes have further indicated the differences in accelerometer-based or self-report measures.

Interestingly, there appear to be clear and distinct differences among domains of physical activity. Our study revealed that higher levels of work-domain physical activity were associated with higher likelihood of psychological distress using the K6, while transport-domain physical activity was not associated with psychological distress. Whether the relationship between higher physical activity levels and higher psychological distress was related to the different amount of activity time occurred in each domain remains unclear. However, when compared with other studies that assessed the associations of domain-specific physical activity with psychological health, it did not appear to be the case that our population engaged in a particularly higher amount of work-related physical activity or lower amount of leisure-time physical activity [60, 61].

Higher levels of leisure-time physical activity, however, were consistently associated with less psychological distress using both the K6 and GHQ-12. Few previous studies have examined psychological health indicators with domain-specific physical activity. A large multi-country study of 11,637 adults from the Eurobarometer 2002 data suggested strong associations for domestic-, occupational-, and leisure-time domains of physical activity with happiness (assessed using the Short Form Health Survey, SF-36); but not for transport-domain physical activity [62]. Mason et al. [63] assessed a sample of 4059 British adults from deprived neighbourhoods in Glasgow and found that physical activity in all domains ameliorated mental health and well-being using the Warwick-Edinburgh Mental Wellbeing Scale. Our study corroborated earlier findings from a meta-analysis that has specifically looked into studies that reported physical activity across various domains and psychological health [35]. White et al. [35] showed that leisure-time physical activity has a stronger relationship with better psychological health, and that work-domain physical activity was associated with worse psychological health. The performance of physical activity during leisure-time may provide a sense of autonomy and distraction from stress [64]. The lack of an association between transport-domain physical activity and psychological distress has also been observed in a study of 2194 Australian adults [60]. These findings may be attributed to the undertaking of active commuting which may be less enjoyable as it is often used as a means of getting to work, train stations or bus stops by walking which has a low-intensity and is often solitary. It is unclear whether the difference in the associations between leisure-time and work-domain physical activities for psychological distress can be attributed to the different intensity of the activities engaged in these domains or merely to the difference in the type of activities. It is possible that work-domain physical activity was not perceived as a self-controlled behaviour or choice of task performance. Also, the performance of work-related physical activity has been linked to symptoms of lower back pain or other associated negative physical health outcomes [65]. This collection of evidence shows that differences in the associations between psychological distress and domain-specific physical activity do exist, implying that psychological distress is influenced by the context in which physical activity is performed. Engagement in leisure-time physical activity should thus be encouraged and could be an effective means of health promotion strategy.

Self-reported total sedentary behaviour was not associated with psychological distress, and this was consistent across the K6 and the GHQ-12. This interpretation contrasts with that of previous studies which showed detrimental associations between self-reported sedentary behaviour and psychological distress using the GHQ-12 [31, 33] and the K10 [66]. We also did not find any association between self-reported domain-specific sedentary behaviour and psychological distress. To date, the limited evidence has generally been mixed. For example, Proper et al.’s study [34] found no association between work-domain sedentary behaviour and mental health, but not in Kilpatrick et al.’s study [66]. Also, Hamer et al. [33] found an equivocal association of TV viewing time on psychological distress, but not in Atkin et al.’s study [20]. Differences in the use of dissimilar self-report indicators of total sedentary behaviour could underlie some of the inconsistencies in results. In Hamer et al.’s study [33], for instance, sedentary behaviour questionnaire with items on TV viewing and other leisure-time sitting such as reading and computer use was employed. These aspects of our results should always be confirmed by further investigations.

Our study found that accelerometer-based total sedentary behaviour was associated with higher likelihood of psychological distress. The present findings were consistent with previous studies which included accelerometer measurement of sedentary behaviour. Hamer et al. [33] showed that higher levels of accelerometer-based sedentary behaviour were also associated with psychological distress using the GHQ-12. Likewise, the NHANES 2005–2006 revealed that higher levels of accelerometer-based sedentary behaviour were associated with a higher likelihood of depression, although the association was not statistically significant in the fully adjusted model [56]. In a smaller study, lower levels of accelerometer-based sedentary behaviour (< 8 h/day) have also been associated with fewer depressive symptoms in sedentary, overweight and obese U.S. women [67]. The associations between higher levels of accelerometer-based sedentary behaviour and higher psychological distress may reflect the effect of being sedentary on one’s development of social networks [68]. This finding again highlights the importance of describing the association between psychological distress and sedentary behaviour (and physical activity) assessed using accelerometer measurement.

The strengths of the present study include the assessment of accelerometer-based and self-reported physical activity and sedentary behaviour, and different psychological measures. This study adds strength to previous studies in assessing self-reported physical activity and sedentary behaviour across various life domains. Also, the study was conducted in a fairly large nationally representative sample of an urban Asian population consisting of men and women from a wide range of educational and socioeconomic backgrounds.

Nonetheless, limitations of the study need to be considered, too. First, the cross-sectional design precludes inferences about the direction of the association between physical activity/ sedentary behaviour and psychological distress. For instance, it was unclear if individuals with psychological distress may have difficulties in being physically active. Second, the domain-specific sedentary behaviour questionnaire has yet to be validated in our population, but unpublished data shows fair-to-moderate correlations with the GPAQ single-item sitting (*r* = 0.58; *p* < 0.001) and accelerometry (*r* = 0.28; *p* < 0.001), which is consistent with the findings from a recent review of sedentary behaviour questionnaires [69]. Third, the waist-worn ActiGraph GT3X accelerometer may not be the most precise measurement of objective physical activity because of its inability to capture physical activities that are static (e.g. weightlifting, carrying heavy loads, etc.) or involve only upper body movement [70]. The ActiGraph also has limited utility in accurately distinguishing between standing still and sitting down. However, it has been validated in the lab [54] and field settings [55] for the measures of physical activity, and a validated cut-point of 150 CPM has been employed to define sedentary behaviour. Although manual visual inspection of accelerometry-based sleep periods has also been employed as a reference method to develop automated algorithms in previous studies [71–73], this may have inadvertently introduced measurement error; hence, readers should be aware of the potential limitations of study findings. Future studies may compare self-reported domain-specific physical activity and sedentary behaviour against time-stamped accelerometer-based data for a more precise assessment of the different contexts of physical activity and sedentary behaviour.

## Conclusion

In conclusion, our findings demonstrated that more leisure-time physical activity and less sedentary behaviour were associated with reduced psychological distress in an urban Asian setting, whereas higher levels of work-domain or transport physical activity were not associated with less psychological distress. Higher levels of work-domain physical activity were associated with higher levels of psychological distress. To improve psychological health, interventions could promote leisure-time physical activity as a targeting construct. This study underscores the importance of assessing accelerometer-based and self-reported domain-specific activity in relation to mental health, instead of solely focusing on total volume of activity.

## Additional file

Additional file 1: **Table S1.** Participants’ characteristics according to K6 psychological distress scale in SH2 study and physical activity sub-study. **Table S2.** Participants’ characteristics according to GHQ-12 psychological distress scale in SH2 study and physical activity sub-study. **Table S3.** Domain-specific physical activity by self-report in relation to psychological distress in subsample (*n* = 703). (DOCX 88 kb)

## Abbreviations

BMI: Body mass index
CPM: Counts per min
GHQ-12: Health Questionnaire
GPAQ: Physical Activity Questionnaire
IQR: Interquartile range
K6: Kessler screening scale
MVPA: Moderate-to-vigorous intensity physical activity
NHANES: National Health and Nutrition Examination Survey
OR: Odds ratio
SH2: Singapore Health 2
VIF: Variance inflation factor
WHO: World Health Organization
