# Supplementary material for: Self-reported domain-specific and accelerometer-based physical activity and sedentary behaviour in relation to psychological distress among an urban Asian population
Source: Int J Behav Nutr Phys Act. 2018 Apr 5;15:36. doi: 10.1186/s12966-018-0669-1 (PMC5885357; doi:10.1186/s12966-018-0669-1)
Supplement: Supplementary file 1 — Table S1. Participants’ characteristics according to K6 psychological distress scale in SH2 study and physical activity sub-study. Table S2. Participants’ characteristics according to GHQ-12 psychological distress scale in SH2 study and physical activity sub-study. Table S3. Domain-specific physical activity by self-report in relation to psychological distress in subsample (n = 703). (DOCX 88 kb) [file 12966_2018_669_MOESM1_ESM.docx]

**Table S1. Participants’ characteristics according to K6 psychological distress scale in SH2 study and physical activity sub-study**

|  | **Overall sample (n=2653)** | |  | |  | **Subsample (n=703)** | |  | |  |
| --- | --- | --- | --- | --- | --- | --- | --- | --- | --- | --- |
|  | **Mod-to-high distress (n=224)** | | **No-to-low distress  (n=2429)** | | **p-value*** | **Mod-to-high distress  (n=69)** | | **No-to-low distress  (n=634)** | | **p-value*** |
|  | **n** | **(%)** | **n** | **(%)** |  | **n** | **(%)** | **n** | **(%)** |  |
| Age (Med, IQR) | 40.0 (26.0-50.0) |  | 46.0 (35.0-58.0) |  | <0.001 | 35.0 (25.0-47.0) |  | 46.0 (35.0-57.0) |  | <0.001 |
| Gender |  |  |  |  | 0.17 |  |  |  |  | 0.91 |
| Men | 91 | 59.4 | 1102 | 45.4 |  | 41 | 59.4 | 377 | 59.5 |  |
| Women | 133 | 40.6 | 1327 | 54.6 |  | 28 | 40.6 | 257 | 40.5 |  |
| Ethnicity |  |  |  |  | <0.001 |  |  |  |  | 0.006 |
| Chinese | 126 | 56.3 | 1644 | 67.7 |  | 34 | 49.3 | 435 | 68.6 |  |
| Indian | 46 | 20.5 | 356 | 14.6 |  | 16 | 23.2 | 81 | 12.8 |  |
| Malay | 48 | 21.4 | 339 | 14.0 |  | 17 | 24.6 | 93 | 14.7 |  |
| Others | 4 | 1.8 | 90 | 3.7 |  | 2 | 2.9 | 25 | 3.9 |  |
| Marital status |  |  |  |  | <0.001 |  |  |  |  | <0.001 |
| Not married ^a^ | 120 | 53.6 | 832 | 34.3 |  | 43 | 62.3 | 232 | 36.6 |  |
| Married | 104 | 46.4 | 1597 | 65.8 |  | 26 | 37.7 | 402 | 63.4 |  |
| Education level |  |  |  |  | 0.14 |  |  |  |  | 0.03 |
| Secondary & below | 65 | 29.1 | 735 | 30.3 |  | 18 | 26.1 | 143 | 22.6 |  |
| Pre-tertiary | 115 | 51.3 | 1095 | 45.1 |  | 42 | 60.9 | 314 | 49.5 |  |
| University & above | 44 | 19.6 | 599 | 24.7 |  | 9 | 13.0 | 177 | 27.9 |  |
| Employment status |  |  |  |  | 0.07 |  |  |  |  | 0.04 |
| Unemployed | 46 | 20.5 | 631 | 26.0 |  | 9 | 13.0 | 153 | 24.1 |  |
| Employed | 178 | 79.5 | 1798 | 74.0 |  | 60 | 87.0 | 481 | 75.9 |  |
| BMI, kg/m^2^ (Med, IQR) | 24.3 (20.9-26.6) |  | 24.2 (21.0-26.5) |  | 0.56 | 24.3 (20.9-26.6) |  | 24.2 (21.0-26.5) |  | 0.79 |
| BMI category |  |  |  |  | 0.57 |  |  |  |  | 0.65 |
| ≤22.9 Normal | 105 | 46.9 | 1069 | 42.0 |  | 30 | 43.5 | 261 | 41.2 |  |
| ≥23.0 & ≤27.4 Overweight | 78 | 34.8 | 900 | 37.1 |  | 24 | 34.8 | 255 | 40.2 |  |
| ≥27.5 Obese | 41 | 18.3 | 460 | 18.9 |  | 15 | 21.7 | 118 | 18.6 |  |
| Smoking status |  |  |  |  | 0.06 |  |  |  |  | 0.01 |
| Never smoker | 146 | 65.2 | 1727 | 71.1 |  | 41 | 59.4 | 472 | 74.4 |  |
| Ever smoker | 78 | 34.8 | 702 | 28.9 |  | 28 | 40.6 | 162 | 25.6 |  |
| Alcohol drinking |  |  |  |  | 0.44 |  |  |  |  | 0.55 |
| Non-drinker | 120 | 53.6 | 1313 | 54.0 |  | 32 | 46.4 | 328 | 51.7 |  |
| Irregular drinker | 79 | 35.3 | 905 | 37.3 |  | 30 | 43.5 | 261 | 41.2 |  |
| Regular drinker | 25 | 11.1 | 211 | 8.7 |  | 7 | 10.1 | 45 | 7.1 |  |
| GHQ-12 score (Med, IQR) | 3.0 (1.5-6.0) |  | 0 (0-1.0) |  | <0.001 | 3.0 (2.0-5.0) |  | 0 (0-1.0) |  | <0.001 |
| Asthma | 42 | 18.8 | 239 | 9.8 | <0.001 | 13 | 18.8 | 56 | 8.8 | 0.01 |
| Cancer | 5 | 2.2 | 47 | 1.9 | 0.76 | 1 | 1.5 | 15 | 2.4 | 0.84 |
| Diabetes mellitus | 18 | 8.0 | 217 | 8.9 | 0.65 | 6 | 8.7 | 46 | 7.3 | 0.67 |
| Heart attack | 2 | 0.9 | 32 | 1.3 | 0.59 | 0 | 0 | 9 | 1.4 | 0.30 |
| Stroke | 4 | 1.8 | 27 | 1.1 | 0.78 | 7 | 1.0 | 6 | 1.0 | 0.90 |
| Presence of at least one disease | 60 | 26.8 | 500 | 20.6 | 0.03 | 18 | 26.1 | 118 | 18.6 | 0.14 |

* p-value: Test of significance between psychological distress levels.

^a^ Single, divorced or widowed.

**Table S2. Participants’ characteristics according to GHQ-12 psychological distress scale in SH2 study and physical activity sub-study**

|  | **Overall sample (n=2653)** | |  | |  | **Subsample (n=703)** | |  | |  |
| --- | --- | --- | --- | --- | --- | --- | --- | --- | --- | --- |
|  | **Psychological distress (n=577)** | | **Normal  (n=2076)** | | **p-value*** | **Psychological distress (n=175)** | | **Normal  (n=528)** | | **p-value*** |
|  | **n** | **(%)** | **n** | **(%)** |  | **n** | **(%)** | **n** | **(%)** |  |
| Age (Med, IQR) | 43.0 (31.0-54.0) |  | 46.0 (35.0-59.0) |  | <0.001 | 43.0 (30.0-54.0) |  | 46.0 (35.0-57.5) |  | <0.01 |
| Gender |  |  |  |  | 0.14 |  |  |  |  | 0.48 |
| Men | 244 | 42.3 | 949 | 45.7 |  | 67 | 38.3 | 218 | 41.3 |  |
| Women | 333 | 57.7 | 1127 | 54.3 |  | 108 | 61.7 | 310 | 58.7 |  |
| Ethnicity |  |  |  |  | 0.35 |  |  |  |  | 0.60 |
| Chinese | 383 | 66.4 | 1387 | 66.8 |  | 111 | 63.4 | 358 | 67.8 |  |
| Indian | 83 | 14.4 | 319 | 15.4 |  | 29 | 16.6 | 68 | 12.9 |  |
| Malay | 95 | 16.5 | 292 | 14.1 |  | 28 | 16.0 | 82 | 15.5 |  |
| Others | 16 | 2.8 | 78 | 3.8 |  | 7 | 4.0 | 20 | 3.8 |  |
| Marital status |  |  |  |  | <0.001 |  |  |  |  | <0.01 |
| Not married ^a^ | 247 | 42.8 | 705 | 34.0 |  | 85 | 48.6 | 190 | 36.0 |  |
| Married | 330 | 57.2 | 1371 | 66.0 |  | 90 | 51.4 | 338 | 64.0 |  |
| Education level |  |  |  |  | 0.70 |  |  |  |  | 0.33 |
| Secondary & below | 166 | 28.8 | 634 | 30.5 |  | 47 | 26.9 | 114 | 21.6 |  |
| Pre-tertiary | 270 | 46.8 | 940 | 45.3 |  | 86 | 49.1 | 270 | 51.1 |  |
| University & above | 141 | 24.4 | 502 | 24.2 |  | 42 | 24.0 | 144 | 27.3 |  |
| Employment status |  |  |  |  | 0.85 |  |  |  |  | 0.73 |
| Unemployed | 149 | 25.8 | 528 | 25.4 |  | 42 | 24.0 | 120 | 22.7 |  |
| Employed | 428 | 74.2 | 1548 | 74.6 |  | 133 | 76.0 | 408 | 77.3 |  |
| BMI, kg/m^2^ (Med, IQR) | 23.4 (20.9-26.6) |  | 23.7 (21.0-26.5) |  | 0.32 | 23.6 (21.0-27.2) |  | 23.8 (21.1-26.4) |  | 0.80 |
| BMI category |  |  |  |  | 0.33 |  |  |  |  | 0.65 |
| ≤22.9 Normal | 271 | 47.0 | 903 | 43.5 |  | 77 | 44.0 | 214 | 40.5 |  |
| ≥23.0 & ≤27.4 Overweight | 201 | 34.8 | 777 | 37.5 |  | 60 | 34.3 | 219 | 41.5 |  |
| ≥27.5 Obese | 105 | 18.2 | 396 | 19.1 |  | 38 | 21.7 | 95 | 18.0 |  |
| Smoking status |  |  |  |  | 0.58 |  |  |  |  | 0.80 |
| Never smoker | 402 | 69.7 | 1471 | 70.9 |  | 129 | 59.4 | 384 | 72.7 |  |
| Ever smoker | 175 | 30.3 | 605 | 29.1 |  | 46 | 40.6 | 144 | 27.3 |  |
| Alcohol drinking |  |  |  |  | 0.04 |  |  |  |  | 0.87 |
| Non-drinker | 299 | 51.8 | 1,134 | 54.6 |  | 62 | 35.4 | 197 | 37.3 |  |
| Irregular drinker | 218 | 37.8 | 766 | 36.9 |  | 99 | 56.6 | 293 | 55.5 |  |
| Regular drinker | 60 | 10.4 | 176 | 8.5 |  | 14 | 8.0 | 38 | 7.2 |  |
| K6 score (Med, IQR) | 11.0 (8.0-14.0) |  | 7.0 (6.0-9.0) |  | <0.001 | 11.0 (8.0-15.0) |  | 7.0 (6.0-9.0) |  | <0.001 |
| Asthma | 85 | 14.7 | 196 | 9.4 | <0.001 | 26 | 14.9 | 43 | 8.1 | 0.01 |
| Cancer | 14 | 2.4 | 38 | 1.8 | 0.73 | 4 | 2.3 | 12 | 2.3 | 0.85 |
| Diabetes mellitus | 52 | 9.0 | 183 | 8.8 | 0.89 | 15 | 8.6 | 37 | 7.0 | 0.49 |
| Heart attack | 9 | 1.6 | 25 | 1.2 | 0.72 | 1 | 0.6 | 8 | 1.5 | 0.34 |
| Stroke | 10 | 1.7 | 21 | 1.0 | 0.50 | 2 | 1.1 | 5 | 0.9 | 0.83 |
| Presence of at least one disease | 144 | 25.0 | 416 | 20.0 | 0.01 | 42 | 24.0 | 94 | 17.8 | 0.07 |

* p-value: Test of significance between psychological distress levels.

^a^ Single, divorced or widowed.

**Table S3. Domain-specific physical activity by self-report in relation to psychological distress in subsample (n=703)**

|  |  | **K6** |  |  |  |  |  | **GHQ-12** |  |  |  |  |  |
| --- | --- | --- | --- | --- | --- | --- | --- | --- | --- | --- | --- | --- | --- |
|  |  | **Model 1^a^** | | | **Model**  **2^b^** | | | **Model 1^a^** | | | **Model**  **2^b^** | | |
| **Physical activity (n=703)** | **n** | **OR** | **95% CI** |  | **OR** | **95% CI** |  | **OR** | **95% CI** |  | **OR** | **95% CI** |  |
| **Work (min/wk)^c^** |  |  |  |  |  |  |  |  |  |  |  |  |  |
| T1 (=0) | 397 | Ref. |  |  | Ref. |  |  | Ref. |  |  | Ref. |  |  |
| T2 (>0 & ≤360.0) | 167 | 1.43 | 0.77 | 2.63 | 1.10 | 0.56 | 2.16 | 1.04 | 0.68 | 1.58 | 0.98 | 0.63 | 1.52 |
| T3 (>360.0) | 139 | 1.98 | 1.09 | 3.61 | 1.97 | 1.00 | 3.93 | 1.08 | 0.69 | 1.68 | 1.04 | 0.63 | 1.70 |
| p for trend |  | 0.02 |  |  | 0.07 |  |  | 0.72 |  |  | 0.07 |  |  |
| **Transport (min/wk)^c^** |  |  |  |  |  |  |  |  |  |  |  |  |  |
| T1 (=0) | 146 | Ref. |  |  | Ref. |  |  | Ref. |  |  | Ref. |  |  |
| T2 (>100.0 & ≤180.0) | 280 | 1.26 | 0.60 | 2.62 | 1.31 | 0.59 | 2.90 | 0.80 | 0.52 | 1.27 | 0.75 | 0.47 | 1.20 |
| T3 (>180.0) | 277 | 1.60 | 0.78 | 3.28 | 1.53 | 0.70 | 3.36 | 0.74 | 0.47 | 1.17 | 0.71 | 0.44 | 1.14 |
| p for trend |  | 0.17 |  |  | 0.28 |  |  | 0.22 |  |  | 0.18 |  |  |
| **Leisure-time (min/wk)^c^** |  |  |  |  |  |  |  |  |  |  |  |  |  |
| T1 (=0) | 319 | Ref. |  |  | Ref. |  |  | Ref. |  |  | Ref. |  |  |
| T2 (>0 & ≤120.0) | 222 | 0.48 | 0.26 | 0.91 | 0.47 | 0.23 | 0.95 | 0.69 | 0.46 | 1.03 | 0.64 | 0.41 | 0.98 |
| T3 (>120.0) | 162 | 0.79 | 0.43 | 1.46 | 0.70 | 0.35 | 1.41 | 0.72 | 0.46 | 1.11 | 0.65 | 0.41 | 1.05 |
| p for trend |  | 0.23 |  |  | 0.21 |  |  | 0.08 |  |  | 0.05 |  |  |

OR, odds ratio; CI, confidence interval.

^a^ Unadjusted odds.

^b^ Adjusted for age, gender, ethnicity, marital status, education, employment status, BMI, smoking status, alcohol drinking, presence of at least 1 disease and sedentary behaviour.

^c^ T1 includes only zero values, T2 and T3 are grouped by median splitting the remaining values.
